# Venous thromboembolism in critically ill COVID-19 patients receiving prophylactic or therapeutic anticoagulation: a systematic review and meta-analysis

**DOI:** 10.1007/s11239-020-02235-z

**Published:** 2020-08-03

**Authors:** Syed Shahzad Hasan, Sam Radford, Chia Siang Kow, Syed Tabish Razi Zaidi

**Affiliations:** 1 School of Applied Sciences, University of Huddersfield, Huddersfield, UK; 2 Intensive Care Unit, Austin Health, Melbourne, Australia; 3 School of Medicine, University of Melbourne, Melbourne, Australia; 4 School of Postgraduate Studies, International Medical University, Kuala Lumpur, Malaysia; 5 School of Healthcare, University of Leeds, Leeds, UK; 6 Leeds Teaching Hospitals Trust, Leeds, UK

**Keywords:** Anticoagulation, Coronavirus 2019, COVID-19, Critically ill, Venous thromboembolism

## Abstract

Many aspects of care such as management of hypercoagulable state in COVID-19 patients, especially those admitted to intensive care units is challenging in the rapidly evolving pandemic of novel coronavirus disease 2019 (COVID-19). We seek to systematically review the available evidence regarding the anticoagulation approach to prevent venous thromboembolism (VTE) among COVID-19 patients admitted to intensive care units. Electronic databases were searched for studies reporting venous thromboembolic events in patients admitted to the intensive care unit receiving any type of anticoagulation (prophylactic or therapeutic). The pooled prevalence (and 95% confidence interval [CI]) of VTE among patients receiving anticoagulant were calculated using the random-effects model. Subgroup pooled analyses were performed with studies reported prophylactic anticoagulation alone and with studies reported mixed prophylactic and therapeutic anticoagulation. We included twelve studies (8 Europe; 2 UK; 1 each from the US and China) in our systematic review and meta-analysis. All studies utilized LMWH or unfractionated heparin as their pharmacologic thromboprophylaxis, either prophylactic doses or therapeutic doses. Seven studies reported on the proportion of patients with the previous history of VTE (range 0–10%). The pooled prevalence of VTE among ICU patients receiving prophylactic or therapeutic anticoagulation across all studies was 31% (95% CI 20–43%). Subgroup pooled analysis limited to studies reported prophylactic anticoagulation alone and mixed (therapeutic and prophylactic anticoagulation) reported pooled prevalences of VTE of 38% (95% CI 10–70%) and 27% (95% CI 17–40%) respectively. With a high prevalence of thromboprophylaxis failure among COVID-19 patients admitted to intensive care units, individualised rather than protocolised VTE thromboprophylaxis would appear prudent at interim.

**Electronic supplementary material:**

The online version of this article (10.1007/s11239-020-02235-z) contains supplementary material, which is available to authorized users.

## Highlights

- Despite receiving anticoagulation for thromboprophylaxis, a high rate of venous thromboembolism was still observed among COVID-19 patients admitted to the intensive care unit.
- The failure rate of pharmacological thromboprophylaxis may be lower with the use of therapeutic anticoagulation among COVID-19 patients admitted to the intensive care unit.
- An individualized dosing approach of anticoagulant based on anti-factor Xa monitoring, thromboelastography, or rotational thromboelastometry may be useful to reduce the rate of venous thromboembolism COVID-19 patients.

## Introduction

The novel coronavirus disease 2019 (COVID-19) has claimed over 500,000 lives and infected well over 9 million people as of 27th June 2020 [1]. COVID-19 infection has demonstrated a range of phenotypes from asymptomatic, all the way to multiorgan failure and death. Among the COVID-19 population admitted to intensive care units (ICUs) there has been considerable reporting of venous thromboembolism (VTE). Though hypercoagulability in COVID-19 has been well-recognized, uncertainty still exists as to how best to manage clotting risk in these patients. Like many aspects of care in this rapidly evolving pandemic, the evidence is scarce with adequate quality to inform the approach to the hypercoagulable state in COVID-19 patients.

Since the recognition of the hypercoagulable state in COVID-19 patients, several interim guidance documents have recommended the use of pharmacologic thromboprophylaxis in hospitalized patients with COVID-19 [2–4]. Most of these guidelines [2–4] recommend the use of unfractionated heparin (UFH) or low molecular weight heparin (LMWH), though the consensus is yet to reach in the recommendations of prophylactic, intermediate, or therapeutic (full) dose anticoagulation. Nevertheless, it is not fully understood how effective pharmacologic thromboprophylaxis in preventing VTE among COVID-19 critically ill patients. We seek to systematically review the available evidence to help guide clinicians weighing up decisions regarding the anticoagulation approach for COVID-19 patients admitted to intensive care units.

## Methods

This systematic review with meta-analysis was conducted with adherence to the Preferred Reporting Items for Systematic Reviews and Meta-analyses (PRISMA) guidelines [5]. Two authors (CSK and SSH) independently performed a systematic literature search in PubMed, and Google Scholar and two preprint servers (medRxiv and SSRN) up to 25th June 2020. Search terms are depicted in Table S1. The titles and abstracts of the resulting articles were examined to exclude irrelevant studies. The full texts of the remaining articles were read to determine if these articles meet our eligibility criteria. Bibliographies of retrieved articles were also reviewed for additional studies. The studies eligible for inclusion reported on the prevalence of venous thromboembolic event (deep vein thrombosis and pulmonary embolism) in patients admitted to the intensive care unit (ICU) receiving any type of anticoagulation (prophylactic or therapeutic). Articles were excluded if they consist of no original data, report combined arterial and venous thromboembolic events, report mixed ICU and medical patients, or report no pharmacologic thromboprophylaxis. Case study, case series, and case report that may not reflect the true prevalence of VTE were also excluded.

Two authors (CSK and SSH) independently reviewed the primary studies to assess the appropriateness for inclusion and data were extracted. Any discrepancies were addressed by a joint re-evaluation of the original article. The information extracted from each study included the name of the first author, the country from which the study was reported, the design of the study, age information of patients, the proportion of patients with a reported previous history of VTE, information on body weight or body mass index, information on the anticoagulant regimen, and the proportion of patients who developed VTE.

The outcome measure was the prevalence of patients receiving anticoagulants who developed VTE from individual studies. The pooled prevalence (and 95% confidence interval [CI]) of VTE among patients receiving anticoagulant were calculated using the random-effects model. Subgroup pooled analyses were performed with studies reported prophylactic anticoagulation alone and with studies reported mixed prophylactic and therapeutic anticoagulation. All meta-analytical calculations were performed using Meta XL, version 5.3 (EpiGear International, Queensland, Australia) [6]. We examined the heterogeneity between studies using the *I*^*2*^ statistics with 50% as the threshold for statistically significant heterogeneity.

## Results

**Fig. 1:**
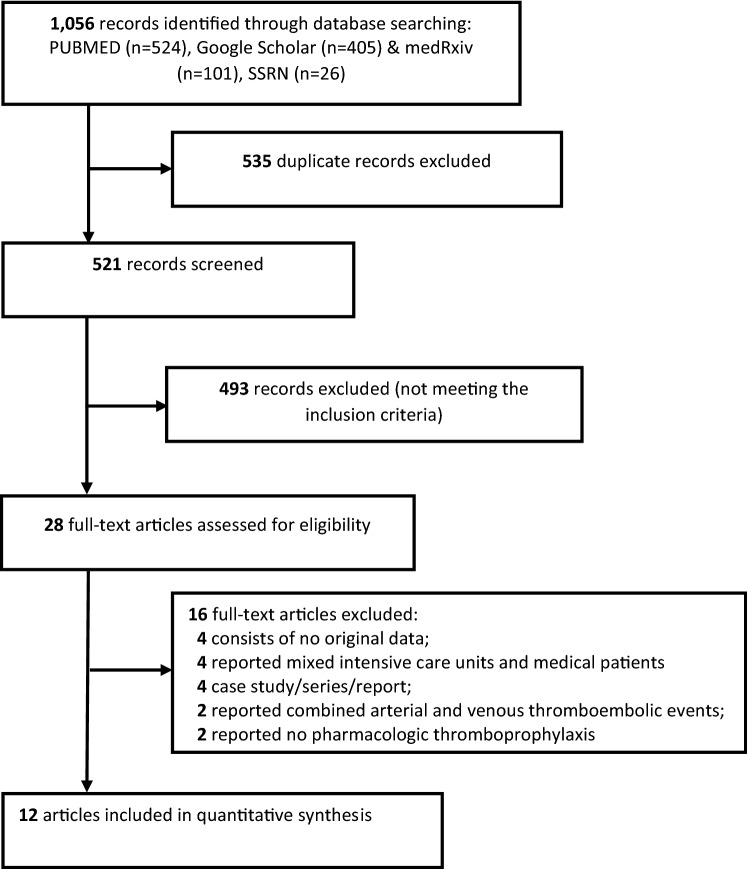
Study selection process (PRISMA)

Our search yielded 1,056 titles from the selected databases (Fig. 1), of which 535 titles were duplicates. The remaining 521 records were screened as per PRISMA guidelines against the eligibility criteria described in the previous section. A total of 493 records were excluded after reading the title and abstract. The full-texts of the remaining 28 articles were retrieved for a detailed evaluation. Sixteen articles were excluded due to reasons including absence of original data, reported mixed intensive care units and medical patients, case study/series/report, reported combined arterial and venous thromboembolic events and reported no pharmacologic thromboprophylaxis.

We eventually identified twelve studies [7–18] that reported the prevalence of VTE among ICU patients receiving prophylactic or therapeutic anticoagulation: three studies from France [7–9], two studies each from the Netherlands [10, 11], Italy [12, 13], the United Kingdom [14, 15], and one study each from the United States [16], China [17], and Germany [18]. The information extracted from each study is presented in Table 1. The number of patients admitted to intensive care units ranged widely from 20 to 184. Eight out of eleven studies used a retrospective chart review as their study design [7, 9–16], whereas among the remaining three studies, two reported prospectively enrolled cohort [8, 18] and one reported a cross-sectional analysis [17]. The mean/median age of patients ranged from 59 to 70 years old. Though not all studies reported on the proportion of patients with the previous history of VTE, we observed a low proportion (0-5.3%) in studies [7–10, 12, 14] which reported the figures except the study by Zerwes et al. [18] which reported a proportion of 10.0%. All studies [7–18] utilized LMWH or unfractionated heparin as their pharmacologic thromboprophylaxis approach, though with a mixed proportion of patients receiving either prophylactic doses or therapeutic doses.

**Table 1:** Summary of studies reporting on the proportion of VTE among COVID-19 patients in ICU receiving anticoagulation

Study	Country	Study design	Mean/median age (y)	Proportion of patients with previous VTE (%)	Body weight/ BMI	Anticoagulant regimen	Proportion of patients who developed VTE (n/N)
Llitjos et al. [7]	France	Retrospective, multicenter	68	3.8	No mention	LMWH or UFH Prophylactic anticoagulation: 31.0% Therapeutic anticoagulation: 69.0%	18/26; 69.2%
Helms et al. [8]	France	Prospective, multicentre	63^1^	5.3	No mention	LMWH or UFH Prophylactic anticoagulation: 70.0% Therapeutic anticoagulation: 30.0%	27/150; 18.0%
Fraissé et al. [9]	France	Retrospective, single-center	61	5.4	Median BMI: 30 kg/m^2^	No mention of anticoagulant Prophylactic anticoagulation: 46.7% Therapeutic anticoagulation: 53.3%	19/92; 20.6%
Middledorp et al. [10]	Netherlands	Retrospective, single-center	62	2.8	Median BMI: 27 kg/m^2^; 17% of patients with body weight ≥ 100 kg	LMWH Both prophylactic and therapeutic anticoagulationwere utilized though no breakdown on prophylactic vs. therapeutic anticoagulation was provided	35/75; 46.7%
Klok et al. [11]	Netherlands	Retrospective, multicenter	64	No mention	Mean body weight: 87 kg	LMWH Prophylactic anticoagulation: 90.8% Therapeutic anticoagulation: 9.2%	28/184; 15.2%
Lodigiani et al. [12]	Italy	Retrospective, single-center	61	0	22.9% of patients with BMI ≥ 30 kg/m^2^	LMWH Prophylactic anticoagulation: 95.8% Therapeutic anticoagulation: 4.2%	8/48; 16.7%
Spiezia et al. [13]	Italy	Retrospective, single-center	67	No mention	Mean BMI: 30 kg/m^2^	LMWH No breakdown on prophylactic vs. therapeutic anticoagulation was provided	5/22; 22.7%
Thomas et al. [14]	United Kingdom	Retrospective, single-center	59	1.6	80.9% of patients with body weight between 50–99 kg	LMWH Prophylactic anticoagulation: 100.0%	17/62; 27.4%
Desborough et al. [15]	United Kingdom	Retrospective, single-center	59	No mention	Median BMI: 28 kg/m^2^	LMWH Prophylactic anticoagulation: 100.0%	10/66; 15.2%
Maatman et al. [16]	United States	Retrospective, multicenter	61	No mention	Mean BMI: 34.8 kg/m^2^	LMWH or UFH Prophylactic anticoagulation: 100.0%	29/107; 27.1%
Ren et al. [17]	China	Cross-sectional, multicentre	70	No mention	33.3% of patients with BMI ≥ 24 kg/m^2^	LMWH Prophylactic anticoagulation: 100.0%	41/47; 87.2%
Zerwes et al. [18]	Germany	Prospective, single-centre	64	10.0	Mean BMI: 28.1 kg/m^2^	LMWH or UFH Both prophylactic and therapeutic anticoagulationwere utilized though no breakdown on prophylactic vs. therapeutic anticoagulation was provided	4/20; 20.0%

*BMI* body mass index, *LMWH* Low molecular weight heparin, *UFH* unfractionated heparin, *VTE* venous thromboembolism

**Fig. 2:**
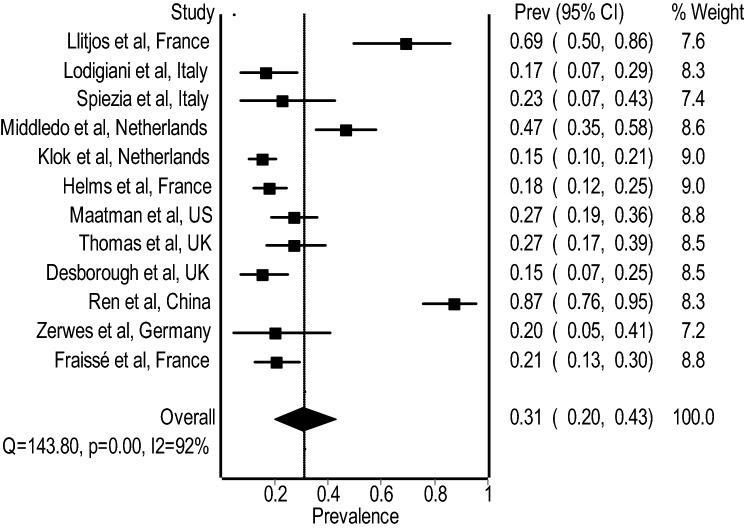
Pooled VTE prevalence (%) in COVID-19 patients admitted to ICU receiving anticoagulation

The pooled prevalence of VTE among ICU patients receiving prophylactic or therapeutic anticoagulation across all studies was 31% (95% CI 20–43%; I2: 92%) (Fig. 2). Subgroup pooled analysis limited to studies reported prophylactic anticoagulation alone in all patients included [14–17] reported a pooled prevalence of VTE of 38% (95% CI 10–70%; I2: 96%) (Supplementary Fig. S1). Subgroup pooled analysis limited to studies reported mixed therapeutic and prophylactic anticoagulation in all patients included [7–13, 18] reported a pooled prevalence of VTE of 27% (95% CI 17–40%; I2: 89%) (Supplementary Fig. S2).

## Discussion

The hypercoagulable state in COVID-19, which has been termed thrombo-inflammation or COVID-19-associated coagulopathy (CAC) by some experts, can be explained in terms of Virchow’s triad: endothelial injury, stasis of blood flow, and hypercoagulability. In terms of endothelial injury, there is evidence of direct invasion of endothelial cells by the SARS-CoV-2 virus, potentially leading to cell injury [19]. Other sources of endothelial injury may include intravascular catheters and mediators of the acute systemic inflammatory response such as cytokines (e.g., interleukin-6) other acute phase reactants [20]. On the other hand, immobilization during hospitalization with COVID-19, especially severely ill patients admitted to intensive care units, can cause stasis of blood flow. In terms of hypercoagulability, many changes in circulating prothrombotic factors have been reported or proposed in patients with severe COVID-19, including elevated factor VIII level, elevated fibrinogen level, circulating prothrombotic microparticles, and neutrophil extracellular traps [21–23].

Therefore, with the recognition of hypercoagulability in COVID-19, the need for effective thromboprophylaxis cannot be overstated. Since no head-to-head trial comparing pharmacologic thromboprophylaxis versus no pharmacologic thromboprophylaxis among ICU patients, as of the time of writing (doubt such trial will be conducted), a comparison of our findings (Table 1) with non-COVID-19 critical care patients is worth exploring. The landmark PROphylaxis for ThromboEmbolism in Critical care (PROTECT) trial that compared the comparative effectiveness of LMWH and UFH in 3764 critically ill patients found the average incidence of VTE of 8.2% and 9.9%, respectively [24]. A subsequent analysis of the PROTECT study by Lim et al. found an overall incidence of VTE of 7.7% [25]. A more relevant comparison of our findings is with critically ill patients who developed sepsis during their hospitalisation, and thus may be at a higher risk of VTE prophylaxis failure. The incidence of VTE in sepsis patients admitted to ICU ranged from 12.5% in a retrospective study of 335 patients to 37% in a prospective study of 113 patients [26, 27]. it is important to note that in the aforementioned prospective study, the average body mass index (BMI) of included patients was 31.7 kg/m^2^ and high BMI is a known factor of VTE as noted in the PROTECT sub-analysis [25].

Since preliminary evidence indicating a possibility of a higher rate of pharmacologic thromboprophylaxis failure in ICU patients with COVID-19 compared to their non-COVID-19 counterparts, a reconsideration of the current approach may be needed, including the need to implement individualized VTE prophylaxis. It has previously been demonstrated that prophylactic LMWH dosing is associated with subtherapeutic anti-factor Xa levels in critically ill patients, and therefore an individualized dosing approach based on anti-factor Xa monitoring may be useful in COVID-19 patients [28–31]. Indeed, it has also been recently discovered that prophylactic LMWH dosing was associated with subtherapeutic anti-factor Xa levels among COVID-19 patients admitted to intensive care units. Dutt et al. [32] reported that 95% of COVID-19 patients had sub-therapeutic anti-factor Xa levels despite prophylactic LMWH, a figure which was about 3.5 times higher compared to the patients admitted to medical wards (27%).

The outcomes of individual adjustment of LMWH dosing guided by anti-factor Xa monitoring are encouraging, though thus far it was only investigated in surgical or trauma patients [33, 34]. The lower pooled prevalence of VTE in studies reported mixed anticoagulation approach (prophylactic and therapeutic) compared to studies reported prophylactic anticoagulation only (27% versus 38%) may have been possibly driven by on average higher rate of attaintment of target anti-factor Xa levels due to the use of therapeutic anticoagulation in some included patients. Nevertheless, sub-therapeutic anti-factor Xa levels have also been observed in COVID-19 patients on twice-daily therapeutic LMWH regimens [35].

Similarly, anti-factor Xa monitoring in patients receiving unfractionated heparin is associated with better attainment of therapeutic anticoagulation compared to activated partial thromboplastin time monitoring whereby it shortens the time to reach the therapeutic range as well as improves the length of time in the therapeutic range [36]. In fact, the phenomenon of heparin resistance has been observed up to 80% of COVID-19 patients treated with unfractionated heparin in which there was a need for high doses of unfractionated heparin (more than 35,000 IU/day) to achieve the target activated partial thromboplastin time presumably due to increased factor VIII levels [37]. Patients requiring high doses of unfractionated heparin to achieve the target activated partial thromboplastin time may also develop life-threatening bleeding events if they proceed without monitoring of antithrombotic activity via an anti-factor Xa assay.

There has been an increased interest to utilize thromboelastography (TEG) or rotational thromboelastometry (ROTEM) in critically ill COVID-19 patients where both tests may be useful to inform individualized clinical decision-making regarding VTE prophylaxis among COVID-19 patients [38]. Viscoelastic observations with TEG among critically ill COVID-19 patients in the intensive care unit revealed hypercoagulable state with decreased R time and K time as well as elevated fibrinogen activity greater than a 73° angle and maximum amplitude more than 65 mm with heparinase correction [39, 40]. Whereas, viscoelastic observations with ROTEM observed significantly higher maximum clot firmness and clotting time as well as significantly shorter clot formation time among COVID-19 patients compared with healthy controls (p < 0.001) [41]. In fact, comparison among COVID-19 patients reported that maximum clot firmness and clotting time were significantly longer in those admitted to medical wards relative to those in specialized wards (intermediate wards/intensive care units). Thus far, there is only one randomized controlled trial [42] which assessed TEG-based protocol for the dosing of unfractionated heparin among patients receiving extracorporeal membrane oxygenation which reported reduced dose of heparin used compared with aPTT-based protocol, with no difference between the two protocols in terms of thrombotic and haemorrhagic events.

Our analysis does have some limitations. Firstly, there were no randomized controlled trials available that investigate the effectiveness of heparin-based pharmacological thromboprophylaxis among critically ill COVID-19 patients at the time of the literature search. Secondly, ten out of twelve studies included in our meta-analysis originate from European countries, which may limit the generalizability of the results to COVID-19 populations from other continents. Thirdly, we were unable to perform subgroup analysis strictly on patients receiving therapeutic anticoagulation and patients receiving prophylactic anticoagulation since the included original studies did not segregate their data based on the intensity of anticoagulation.

## Conclusions

Further study into anticoagulant selection, dosing regimens, and monitoring are needed in this important population of critically ill COVID-19 patients admitted to intensive care units. Until prospective or randomised studies with a clear description of baseline factors and adequate follow up, the best approach for managing VTE will be uncertain. Individualised rather than protocolised thromboprophylaxis would appear prudent at interim. Besides, maintaining a strong index of suspicion for VTE and the possibility of chemoprophylaxis failure is recommended. Likewise, future studies may investigate the effectiveness of anti-factor Xa-guided or TEG/ROTEM-based heparin dosing in reducing the high prevalence of thromboprophylaxis failure in COVID-19 patients.

## Electronic supplementary material

Below is the link to the electronic supplementary material.

Electronic supplementary material 1 (DOCX 39 kb)

## References

[1] Dong E, Du H, Gardner L (2020) An interactive web-based dashboard to track COVID-19 in real time. Lancet Infect Dis. https://coronavirus.jhu.edu/map.html. Accessed 27 June 2020 10.1016/S1473-3099(20)30120-1 PMC7159018 32087114

[2] Zhai Z, Li C, Chen Y (2020). Prevention and treatment of venous thromboembolism associated with coronavirus disease 2019 infection: A Consensus Statement before Guidelines. Thromb Haemost.

[3] Bikdeli B, Madhavan MV, Jimenez D (2020). COVID-19 and thrombotic or thromboembolic disease: implications for prevention, antithrombotic therapy, and follow-up: JACC state-of-the-art review. J Am Coll Cardiol.

[4] Thachil J, Tang N, Gando S (2020). ISTH interim guidance on recognition and management of coagulopathy in COVID-19. J Thromb Haemost.

[5] Moher D, Liberati A, Tetzlaff J, Altman DG, PRISMA Group (2009). Preferred reporting items for systematic reviews and meta-analyses: the PRISMA statement. PLoS Med.

[6] MetaXL Version 5.3 (2019) https://www.epigear.com/index_files/metaxl.html. Accessed 27 June 2020

[7] Llitjos JF, Leclerc M, Chochois C (2020). High incidence of venous thromboembolic events in anticoagulated severe COVID-19 patients. J Thromb Haemost.

[8] Helms J, Tacquard C, Severac F (2020). High risk of thrombosis in patients with severe SARS-CoV-2 infection: a multicenter prospective cohort study. Intensive Care Med.

[9] Fraissé M, Logre E, Pajot O, Mentec H, Plantefève G, Contou D (2020). Thrombotic and hemorrhagic events in critically ill COVID-19 patients: a French monocenter retrospective study. Crit Care.

[10] Middeldorp S, Coppens M, van Haaps TF (2020). Incidence of venous thromboembolism in hospitalized patients with COVID-19. J Thromb Haemost.

[11] Klok FA, Kruip MJHA, van der Meer NJM (2020). Incidence of thrombotic complications in critically ill ICU patients with COVID-19. Thromb Res.

[12] Lodigiani C, Iapichino G, Carenzo L (2020). Venous and arterial thromboembolic complications in COVID-19 patients admitted to an academic hospital in Milan, Italy. Thromb Res.

[13] Spiezia L, Boscolo A, Poletto F (2020). COVID-19-related severe hypercoagulability in patients admitted to intensive care unit for acute respiratory failure. Thromb Haemost.

[14] Thomas W, Varley J, Johnston A (2020). Thrombotic complications of patients admitted to intensive care with COVID-19 at a teaching hospital in the United Kingdom. Thromb Res.

[15] Desborough MJR, Doyle AJ, Griffiths A, Retter A, Breen KA, Hunt BJ (2020). Image-proven thromboembolism in patients with severe COVID-19 in a tertiary critical care unit in the United Kingdom. Thromb Res.

[16] Maatman TK, Jalali F, Feizpour C (2020). Routine venous thromboembolism prophylaxis may be inadequate in the hypercoagulable state of severe coronavirus disease. Crit Care Med.

[17] Ren B, Yan F, Deng Z (2020). Extremely high incidence of lower extremity deep venous thrombosis in 48 patients with severe COVID-19 in Wuhan. Circulation..

[18] Zerwes S, Hernandez Cancino F, Liebetrau D et al (2020) Erhöhtes Risiko für tiefe Beinvenenthrombosen bei Intensivpatienten mit CoViD-19-Infektion? – Erste Daten [Increased risk of deep vein thrombosis in intensive care unit patients with CoViD-19 infections?-Preliminary data] [published online ahead of print, 2020 Jun 5]. Chirurg. 1–7 10.1007/s00104-020-01222-7 PMC7274071 32504106

[19] Varga Z, Flammer AJ, Steiger P (2020). Endothelial cell infection and endotheliitis in COVID-19. Lancet.

[20] Begbie M, Notley C, Tinlin S, Sawyer L, Lillicrap D (2000). The Factor VIII acute phase response requires the participation of NFkappaB and C/EBP. Thromb Haemost.

[21] Panigada M, Bottino N, Tagliabue P et al. (2020) Hypercoagulability of COVID-19 patients in Intensive Care Unit. A Report of Thromboelastography Findings and other Parameters of Hemostasis. J Thromb Haemost. doi:10.1111/jth.14850 10.1111/jth.14850 PMC9906150 32302438

[22] Ranucci M, Ballotta A, Di Dedda U (2020). The procoagulant pattern of patients with COVID-19 acute respiratory distress syndrome. J Thromb Haemost..

[23] Maier CL, Truong AD, Auld SC, Polly DM, Tanksley CL, Duncan A (2020). COVID-19-associated hyperviscosity: a link between inflammation and thrombophilia?. Lancet.

[24] Cook D, Meade M, PROTECT Investigators for the Canadian Critical Care Trials Group and the Australian and New Zealand Intensive Care Society Clinical Trials Group (2011). Dalteparin versus unfractionated heparin in critically ill patients. N Engl J Med.

[25] Lim W, Meade M, Lauzier F (2015). Failure of anticoagulant thromboprophylaxis: risk factors in medical-surgical critically ill patients. Crit Care Med.

[26] Hanify JM, Dupree LH, Johnson DW, Ferreira JA (2017). Failure of chemical thromboprophylaxis in critically ill medical and surgical patients with sepsis. J Crit Care.

[27] Kaplan D, Casper TC, Elliott CG (2015). VTE Incidence and Risk Factors in Patients With Severe Sepsis and Septic Shock. Chest.

[28] Priglinger U, Delle Karth G, Geppert A (2003). Prophylactic anticoagulation with enoxaparin: Is the subcutaneous route appropriate in the critically ill?. Crit Care Med.

[29] Rommers MK, Van der Lely N, Egberts TC, van den Bemt PM (2006). Anti-Xa activity after subcutaneous administration of dalteparin in ICU patients with and without subcutaneous oedema: a pilot study. Crit Care.

[30] Robinson S, Zincuk A, Strøm T, Larsen TB, Rasmussen B, Toft P (2010). Enoxaparin, effective dosage for intensive care patients: double-blinded, randomised clinical trial. Crit Care.

[31] Mayr AJ, Dünser M, Jochberger S (2002). Antifactor Xa activity in intensive care patients receiving thromboembolic prophylaxis with standard doses of enoxaparin. Thromb Res.

[32] Dutt T, Simcox D, Downey C (2020). Thromboprophylaxis in COVID-19: Anti-FXa—the missing factor?. Am J Respir Crit Care Med.

[33] Karcutskie CA, Dharmaraja A, Patel J (2018). Association of anti-Factor Xa-guided dosing of enoxaparin with venous thromboembolism after trauma. JAMA Surg.

[34] Kramme K, Sarraf P, Munene G (2020). Prophylactic enoxaparin adjusted by anti-factor Xa peak levels compared with recommended thromboprophylaxis and rates of clinically evident venous thromboembolism in surgical oncology patients. J Am Coll Surg.

[35] White D, MacDonald S, Bull T et al (2020) Heparin resistance in COVID-19 patients in the intensive care unit. J Thromb Thrombolysis 1-5 10.1007/s11239-020-02145-0 PMC7242778 32445064

[36] Guervil DJ, Rosenberg AF, Winterstein AG, Harris NS, Johns TE, Zumberg MS (2011). Activated partial thromboplastin time versus antifactor Xa heparin assay in monitoring unfractionated heparin by continuous intravenous infusion. Ann Pharmacother.

[37] Beun R, Kusadasi N, Sikma M, Westerink J, Huisman A (2020). Thromboembolic events and apparent heparin resistance in patients infected with SARS-CoV-2. Int J Lab Hematol.

[38] Rubulotta F, Soliman-Aboumarie H, Filbey K (2020). Technologies to optimize the care of severe COVID-19 patients for healthcare providers challenged by limited resources. Anesth Analg.

[39] Panigada M, Bottino N, Tagliabue P (2020). Hypercoagulability of COVID-19 patients in intensive care unit: A report of thromboelastography findings and other parameters of hemostasis. J Thromb Haemost.

[40] Mortus JR, Manek SE, Brubaker LS (2020). Thromboelastographic results and hypercoagulability syndrome in patients with coronavirus disease 2019 who are critically Ill. JAMA Netw Open.

[41] Almskog L, Wikman A, Svensson J et al. Rotational Thromboelastometry predicts care level in Covid-19. medRxiv; 2020.06.11.20128710

[42] Panigada M, Iapichino E, Brioni G (2018). Thromboelastography-based anticoagulation management during extracorporeal membrane oxygenation: a safety and feasibility pilot study. Ann Intensive Care.

