# Supplementary material for: Venous thromboembolism in critically ill COVID-19 patients receiving prophylactic or therapeutic anticoagulation: a systematic review and meta-analysis
Source: J Thromb Thrombolysis. 2020 Aug 3;50(4):814–21. doi: 10.1007/s11239-020-02235-z (PMC7396456; doi:10.1007/s11239-020-02235-z)
Supplement: Supplementary file 1 — Electronic supplementary material 1 (DOCX 39 kb) [file 11239_2020_2235_MOESM1_ESM.docx]

**Table S1**: Search Terms

| Database | Search term | Result |
| --- | --- | --- |
| PubMed | (heparin or thrombosis or venous thrombo or pulmonary embol) AND (COVID-19 OR novel coronavirus OR severe acute respiratory syndrome OR SARS-CoV-2) Filters: from 2020 – 2020 | 524 |
| Google Scholar | heparin thrombosis venous thrombo pulmonary embolism SARS-CoV-2 "COVID 19" Filters: from 2020 – 2020 | 405 |
| medRxiv | abstract or title "COVID-19" (match all words) and full text or abstract or title "heparin" (match whole all) and posted between "01 Jan, 2020 and 26 June, 2020 | 101 |
| SSRN | heparin | 26 |


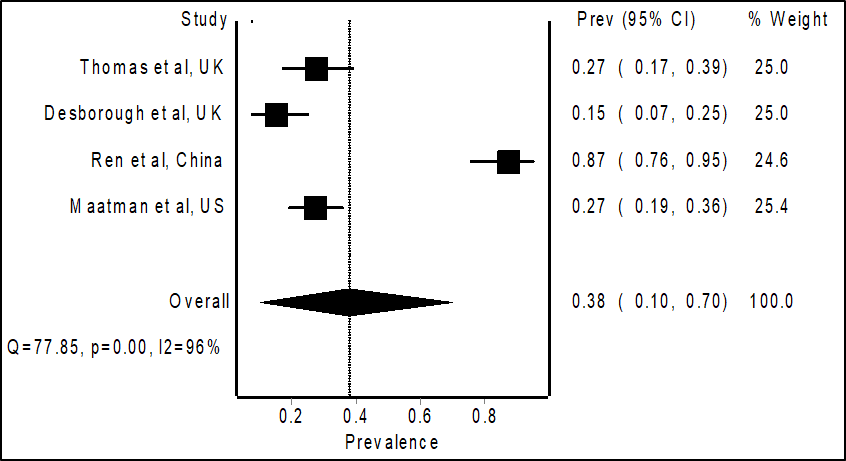


**Figure S1**: Pooled VTE prevalence (%) in COVID-19 patients admitted to ICU receiving prophylactic anticoagulation only

**Figure S2**: Pooled VTE prevalence (%) in COVID-19 patients admitted to ICU receiving either prophylactic or therapeutic anticoagulation
